# Evaluation of the Early Administration of Tranexamic Acid in Geriatric Hip Fractures in the Emergency Department: A Retrospective Study

**DOI:** 10.7759/cureus.62636

**Published:** 2024-06-18

**Authors:** Ralfi Doka, Samuel Neibaur, Saad Mohammad, Roy Small, Michael Dirkx, Amjad Yaish, Grace D Brannan

**Affiliations:** 1 Orthopedic Surgery, McLaren Macomb Hospital, Michigan State University, Mount Clemens, USA; 2 Orthopedic Surgery, McLaren Macomb Hospital, Mount Clemens, USA; 3 Orthopedics and Trauma, University of Pittsburgh Medical Center, Pittsburgh, USA; 4 Orthopedic Surgery, Northwest Iowa Bone, Joint and Sports Surgeons, Spencer, USA; 5 4GDB Research and Statistical Consulting, McLaren Macomb Hospital, Mount Clemens, USA

**Keywords:** trauma and orthopaedic surgery, elderly trauma, quality improvement, hemoglobin, emergency department, hip fracture, tranexamic acid

## Abstract

Introduction

Tranexamic acid (TXA) administration perioperatively has demonstrated efficacy in reducing postoperative drops in hemoglobin levels and the need for transfusions among patients with peritrochanteric hip fractures. This study aims to perform a retrospective analysis to assess the impact on hemoglobin levels by comparing patients with fragility hip fractures who received TXA in the ED, in addition to the standard perioperative TXA dose, with those who did not receive TXA in the ED.

Methods

This study retrospectively reviewed 64 patient records from May 2020 to May 2021 at a Level II trauma center that were classified into two groups: patients who received one gram (g) of TXA in the ED, within five hours of injury (new protocol), or patients who received no TXA in the ED (old protocol). The primary outcomes of the study were hemoglobin and adverse events. An independent t-test was performed on continuous variables. A chi-square test was used to analyze noncontinuous variables. Statistical Product and Service Solutions (SPSS, version 25; IBM SPSS Statistics for Windows, Armonk, NY) was used for analysis. Statistical significance was set at a p value < 0.05.

Results

We measured the difference between hemoglobin on the day of surgery or day zero and on arrival in the ED, which was not statistically significant between the two protocols (p value = 0.322). The difference between hemoglobin levels on postoperative day one and on arrival in the ED was also not statistically significant (p = 0.339). Adverse events were lower in the new protocol but not statistically significant between the two protocols (p = 0.178).

Conclusion

Our study showed improved outcomes in postoperative hemoglobin with early administration of TXA in the ED. This is demonstrated by continuous higher postoperative hemoglobin in the new protocol group without an increase in adverse events. While the data did not achieve statistical significance, we believe there is clinical benefit in the early administration of TXA in the ED, a finding that continues to be explored and supported in the literature.

## Introduction

Despite the advances in orthopedic surgery, the fragility hip fracture mortality rate remains elevated at approximately 30% [1]. These often stem from low-energy falls, with patients having multiple medical comorbidities. These medical comorbidities must be managed throughout the preoperative, intraoperative, and postoperative periods. Preoperative optimization, such as addressing anemia, has been shown to reduce mortality rates [2,3]. Commonly, a 1-3 g decrease in hemoglobin is expected preoperatively from fragility hip fractures [3]. Low preoperative hemoglobin levels have been directly correlated with increased transfusion and mortality rates.

Tranexamic acid (TXA), a pro-coagulant, has been shown to decrease the postoperative drop in hemoglobin [4,5]. TXA is a competitive inhibitor of plasminogen binding sites and prevents the formation of plasmin and subsequent fibrinolysis. The use of TXA is widely accepted in total joint arthroplasty [6]. Preoperative administration of TXA in total joint arthroplasty has been shown to reduce transfusion rates and postoperative wound complications [5,6]. TXA is well tolerated by patients with a low side effect profile and low cost [7]. In the realm of orthopedic trauma, there has been a growing interest in TXA because of its procoagulant benefits.

In peritrochanteric hip fractures, the intraoperative administration of TXA has demonstrated effectiveness in reducing declines in postoperative hemoglobin levels and lowering transfusion rates [8-10]. In a recent study involving elderly patients who underwent hemiarthroplasty for hip fracture, TXA administration before surgery was found to be safe, with higher mean hemoglobin levels, lower rates of transfusion, and no additional risk of deep vein thrombosis compared to those who did not receive TXA [11]. Furthermore, a recent systematic review and meta-analysis found that TXA given intravenously compared to a placebo is safe and efficacious for elderly patients undergoing intramedullary nailing for intertrochanteric hip fractures [12]. Blood loss and the need for blood transfusion were lessened with no elevated risk of adverse events [12]. A broader systematic review and meta-analysis compared TXA with a placebo in the elderly population before surgery and found similar benefits [13].

The Clinical Randomization of an Antifibrinolytic in Significant Hemorrhage 2 (CRASH-2) trial highlighted that the early administration of TXA in trauma patients proved to be a cost-effective method for reducing the risk of death because of blood loss [14]. The intravenous administration of TXA improved mortality rates significantly in trauma patients with significant bleeding. Following the release of its remarkable findings, the conventional treatment protocol for trauma underwent a global transformation, incorporating the administration of TXA [15].

Another finding from the CRASH-2 Trial was the existence of a small window of opportunity to administer TXA [14]. More recent literature reviews have confirmed TXA to be the most effective if given within three hours of the traumatic event [16].

At our level II trauma center, the former protocol for fragility hip fracture patients involved intraoperative administration of 2 grams of TXA, with 1 gram administered at the time of incision and the remaining dose given at the time of closure. However, in response to the compelling evidence, we piloted and revised our treatment protocol to incorporate the early administration of TXA in the ED for patients presenting with fragility hip fractures if identified within five hours of the injury. The purpose of this study was to retrospectively review the effect of the updated treatment protocol.

This article was presented at the 2023 Michigan Summit on Quality Improvement, Patient Safety & High Value Care on May 12, 2023.

## Materials and methods

Study design

This is a single-center retrospective study conducted at McLaren Macomb Hospital, a level II trauma center, Mount Clemens, USA.

Ethical considerations

IRB approval was not required given the retrospective nature of the study. All data were de-identified and obtained by authorized employees (e.g., residents, attendings, research advisors). The study was approved by the McLaren Healthcare System ethics committee (Scholarly Activity Review Committee (SARC) Protocol 202110-02).

Study criteria

Records of patients meeting our inclusion and exclusion criteria were reviewed for the time from May 01, 2020, to May 31, 2021. Patient records were categorized into two groups: The ED TXA group (EDTXA), which received the new treatment protocol, wherein patients received 1 gram (g) of TXA in the ED, and the Non-ED TXA group (NTXA), who received the old treatment protocol, where patients did not receive TXA in the ED. Both groups received 2 g of TXA in the operating room, 1 g at incision, and 1 g at closure. We hypothesized that the implementation of the new protocol would lead to a reduction in the anticipated drop in hemoglobin both preoperatively and postoperatively, ultimately contributing to a decrease in mortality compared to the old protocol. The inclusion criteria for this study were patients > 65 years of age diagnosed with hip fracture (intertrochanteric fracture S72.14 or femoral neck fracture S72.002A ). The exclusion criteria were nondisplaced hip fractures, pathologic hip fractures, fractures resulting from high-energy trauma, polytrauma patients, and patients less than 65 years of age.

Procedure

The demographics, diagnosis, surgical procedure, laboratory results, medicine administration record (MAR), and complications were all obtained via chart review for each patient individually. The time of arrival was determined using the check-in time from the ED records. All data were compiled in an Excel Sheet (Microsoft Corporation, Redmond, WA), confidential only to McLaren organization, and accessible only to employees involved in the study.

Assessment

Hemoglobin was measured at three different times: upon arrival to the ED, on the day of surgery or postoperative day zero, and on postoperative day one. The primary outcomes of the study were differences in perioperative hemoglobin levels measured in grams/deciliter at the three previously identified perioperative time points. Moreover, we measured blood transfusion rates by recording units of blood, platelets, or fresh frozen plasma given perioperatively. The rates of adverse events were also collected. We also measured differences in demographic factors such as age, gender, and type of fracture.

Statistical analyses

Descriptive statistics such as means, frequencies, and percentages were generated, and inferential statistical analyses were performed. For the primary variables, an independent t-test was performed to determine the difference between the EDTXA and NTXA treatment protocols on continuous variables such as hemoglobin levels. A chi-square test was used to analyze categorical variables. Statistical Product and Service Solutions (SPSS, version 25; IBM SPSS Statistics for Windows, Armonk, NY) was used to analyze the data. Statistical significance was set at a p value < 0.05.

## Results

In this study, we analyzed a total of 64 patients with a fragility hip fracture. The EDTXA group consisted of 36 patients, while the NTXA group consisted of 28 patients. The demographics and characteristics of our population are described in Table 1. There was a statistical significance in the mean age (p = 0.008), with the TXA group having a lower mean age, 77 ± 9.3 vs 83.4 ± 9.3. There was not a statistically significant difference between the two groups regarding gender distribution (p = 0.202), race (p = 0.356), or type of fracture (p = 0.899).

**Table 1:** Percentage (frequency) rates or mean (SD) for demographic characteristics of the study population. EDTXA: emergency department tranexamic acid; NTXA: non-emergency department tranexamic acid; SD: standard deviation; N: number of patients Statistical significance set at a P value < 0.05; age is presented as mean ± standard deviation; gender, race, and type of fracture are presented as percentages with the number of patients in parenthesis.

Demographic Factor	Group	Protocol		P value
		EDTXA (new) (N=36)	NTXA (old) (N=28)	
Age (Mean ± SD)		77.00 ± 9.27	83.43 ± 9.34	0.008
Gender	Male	13(36.11%)	6 (21.43%)	0.202
	Female	23 (63.88%)	22 (78.57%)	
Race	1= White	35 (97.22%)	27 (96.43%)	0.356
	2= Black or African American	1 (2.77%)	0 (0.0%)	
	3=Asian	0 (0.0%)	1 (3.57%)	
Type of fracture	Femoral neck	16 (44.44%)	12 (42.86%)	0.899
	Intertrochanteric fractures	20 (55.55%)	16 (57.14%)	

The time from injury (in hours) was statistically significantly higher in the NTXA compared to the EDTXA (17.3 ± 19.8 vs. 2.7 ± 0.7, respectively, p = 0.001). The Glasgow Coma Scale score was 15.0 for both groups. In Table 2, we show the average hemoglobin in the ED, on the day of surgery or postoperative day zero and postoperative day one. No statistically significant difference was identified between the two groups after measuring the hemoglobin levels upon arrival in the ED (p = 0.523), on the day of the surgery or postoperative day zero (p = 0.197) and on postoperative day one (p = 0.301). For the primary outcome, we measured the difference between hemoglobin on the day of the surgery or day zero and in the ED, which was not statistically significant (p = 0.322). The difference between hemoglobin levels on postoperative day one and on arrival in the ED was also not statistically significant (p = 0.339). Notably, the patients in the EDTXA group had higher hemoglobin levels on the day of surgery or postoperative day zero and postoperative day one, and experienced less transfusion, even though these findings were not found to be statistically significant.

**Table 2:** Comparison of different outcomes based on protocol type reported as mean+/- standard deviation. ED: emergency department; *cannot be computed because the standard deviations of both groups are 0. The data were presented as mean ± standard deviation; statistical significance set at a p value < 0.05.

Variable	Protocol		P value
	EDTXA (new) (N=36)	NTXA (Old) (N=28)	
Time from injury (hours)	2.67 ± 0.72	17.29 ± 19.79	0.001
Glasgow Coma Scale (GCS)	15.0	15.0	Not computed*
Hemoglobin levels in ED	12.63 ± 1.31	12.41 ± 1.46	0.523
Hemoglobin levels on the day of surgery or postoperative day 0	11.71 ± 1.56	11.17 ± 1.68	0.197
The difference in hemoglobin day 0 (subtract ED from day of surgery or postoperative day 0)	-0.93 ± 1.34	-1.24 ± 1.06	0.322
Hemoglobin levels postoperative day 1	11.89 ± 1.25	9.42 ± 1.58	0.301
Difference in hemoglobin day 1 (subtract ED from postoperative day 1)	-0.75 ± 12.21	-2.99 ± 12.40	0.339
Amount of transfusion (blood, platelets, fresh frozen plasma)	0.36 ± 0.72	0.43 ± 0.74	0.716

When analyzing adverse events between the two groups, we found no statistically significant difference (p = 0.178) between the EDTXA 33.3% (N = 12) and the NTXA 50% (N = 14) group (Table 3). Any postoperative blood transfusion was recorded as an adverse event for patients in both groups. The rate of blood transfusion was lower in the EDTXA group; however, this was not a statistically significant finding (p = 0.178). The rates for specific adverse events for EDTXA vs NTXA were as follows: rapid response (2.8% (N = 1) vs 3.6% (N = 1), respectively), myocardial infarction (0% (N = 0) vs 3.6% (N = 1), respectively), respiratory complications (0% (N = 0) vs 3.6% (N = 1), respectively), and death (5.6% (N = 2) vs 0% (N = 0), respectively). We did not find stroke or multiorgan failure as an adverse event in either treatment group.

**Table 3:** Adverse events by type of protocol. EDTXA: emergency department tranexamic acid; NTXA: non-emergency department tranexamic acid Adverse event rate includes blood transfusions that occurred in each group. Data were presented as a percentage with a corresponding number of patients in parenthesis; Statistical significance set at a p value <0.05.

Type of Adverse Event	EDTXA (New) (N=36)	NTXA (Old) (N=28)	P value
Adverse event rate	12 (33.33%)	14 (50.0%)	0.178
Rapid response	1 (2.78%)	1 (3.57%)	
Myocardial infarction	0 (0.0%)	1 (3.57%)	
Respiratory complication	0 (0.0%)	3.57% (1)	
Death	2 (5.56%)	1 (0.0%)	

## Discussion

Hip fractures constitute a significant portion of orthopedic healthcare and can have devastating complications, especially in the geriatric population with one-year mortality rates as high as 36% [1]. Our objective was to investigate whether administering TXA early in the ED could reduce the expected decline in hemoglobin levels linked to fragility hip fractures, consequently minimizing the morbidity related to perioperative anemia in elderly patients with hip fractures.

Preoperative hemoglobin is an independent predictor of death, readmission rates, and length of stay in the hospital with higher preoperative hemoglobin being more favorable [3]. This may be because geriatric patients have less physiologic reserve and may be linked to underlying medical comorbidities. Halm et al. found that approximately 40% of patients with hip fractures were anemic on presentation (defined < 12 g/dL) and dropped a mean of 0.3 g/dL preoperatively and 2.8 g/dL after surgery compared to preoperative values [3]. This is different compared to our study wherein the preoperative Hgb drop was 0.9 ± 1.3 g/dL in the TXA group and 1.2 ± 1.1 g/dL in the NTXA group (Table 2).

Many of the current literature, including those studies mentioned previously, compared TXA with a placebo before operation. Our study, in addition to Liu et al. and the CRASH-2 Trial, looked at the early administration of TXA [10,14]. What distinguished our study from existing studies was its comparison of early administration of TXA in addition to perioperative TXA administration, with the sole perioperative administration in patients with fragility hip fractures.

While our study did not show statistical significance, we found the EDTXA group to be trending toward more favorable outcomes. This is demonstrated by a lower hemoglobin drop in patients receiving the new treatment protocol without an increase in adverse events. Furthermore, there has been an increase in literature regarding the early administration of TXA upon admission. Moran et al. found in their prospective analysis a statistical benefit in the early administration of TXA in hip fractures [17]. Another prospective randomized controlled trial showed similar findings in the administration of TXA upon admission; a statistically significant improvement in hemoglobin drops, and transfusion rates [18].

Several limitations to our study could influence our non-statistically significant findings. The small sample size is one limitation. The retrospective nature of this study is also a major limitation. We did not prospectively follow the patients after admission, and all the records were obtained by reviewing their medical records a year later. Given the overwhelming support of TXA in hip fractures, we encourage orthopedic trauma providers to further analyze the effect of early administration of TXA in hip fractures. Further studies with control groups, such as randomized control trials, should prospectively analyze the effects of early administration of TXA.

## Conclusions

There is overwhelming research supporting the use of TXA in fragility hip fractures; however, there are no explicit guidelines regarding the timing of administration. In our study, there were no statistically significant differences in patients with fragility hip fractures who received an early administration of TXA upon admission to the ED in addition to perioperative TXA compared to patients who only received TXA perioperatively. Administration of early TXA did not demonstrate increased side effects in our patient population, a finding that supports the safe side effect profile of TXA.
